# Identification of Healthy Tissue from Malignant Tissue in Surgical Margin Using Raman Spectroscopy in Oral Cancer Surgeries

**DOI:** 10.3390/biomedicines11071984

**Published:** 2023-07-13

**Authors:** Mukta Sharma, Ying-Chang Li, S. N. Manjunatha, Chia-Lung Tsai, Ray-Ming Lin, Shiang-Fu Huang, Liann-Be Chang

**Affiliations:** 1 Department of Electronic Engineering, Chang Gung University, Taoyuan 33302, Taiwan; mukta.shrm@gmail.com (M.S.); lianncgu@gmail.com (L.-B.C.); 2 Department of Ph.D. Program, Prospective Technology of Electrical Engineering and Computer Science, National Chin-Yi University of Technology, Taichung 411030, Taiwan; 3 Department of Otolaryngology-Head and Neck Surgery, Chang Gung Memorial Hospital, Linkou 333, Taiwan; shiangfu.huang@gmail.com; 4 Department of Public Health, Chang Gung University, Taoyuan 33302, Taiwan

**Keywords:** Raman spectroscopy, oral cancer, partial least squares, support vector machine, tissue

## Abstract

Accurate identification of tissue types in surgical margins is essential for ensuring the complete removal of cancerous cells and minimizing the risk of recurrence. The objective of this study was to explore the clinical utility of Raman spectroscopy for the detection of oral squamous cell carcinoma (OSCC) in both tumor and healthy tissues obtained from surgical resection specimens during surgery. This study enrolled a total of 64 patients diagnosed with OSCC. Among the participants, approximately 50% of the cases were classified as the most advanced stage, referred to as T4. Raman experiments were conducted on cryopreserved tissue samples collected from patients diagnosed with OSCC. Prominent spectral regions containing key oral biomarkers were analyzed using the partial least squares–support vector machine (PLS–SVM) method, which is a powerful multivariate analysis technique for discriminant analysis. This approach effectively differentiated OSCC tissue from non-OSCC tissue, achieving a sensitivity of 95.7% and a specificity of 93.3% with 94.7% accuracy. In the current study, Raman analysis of fresh tissue samples showed that OSCC tissues contained significantly higher levels of nucleic acids, proteins, and several amino acids compared to the adjacent healthy tissues. In addition to differentiating between OSCC and non-OSCC tissues, we have also explored the potential of Raman spectroscopy in classifying different stages of OSCC. Specifically, we have investigated the classification of T1, T2, T3, and T4 stages based on their Raman spectra. These findings emphasize the importance of considering both stage and subsite factors in the application of Raman spectroscopy for OSCC analysis. Future work will focus on expanding our tissue sample collection to better comprehend how different subsites influence the Raman spectra of OSCC at various stages, aiming to improve diagnostic accuracy and aid in identifying tumor-free margins during surgical interventions.

## 1. Introduction

Oral cancer, associated with significant morbidity and mortality rates, involves the development of cancerous cells in the tissues of the oral cavity, including the mouth, tongue, lips, gums, the floor of the mouth, and the inner lining of the cheeks [1]. OSCC is the most common type of oral cancer, accounting for approximately 90% of all oral malignancies, which are often detected at a later stage, leading to a five-year survival rate of approximately 50% [2]. Oral potentially malignant disorders (OPMDs) are mucosal irregularities that carry a higher risk of developing into OSCC. Timely detection and intervention are essential to mitigate its life-treatening potential. Annually, around 377,713 new cases are diagnosed, with the highest number (248,360) recorded in Asia; there are 177,757 deaths due to oral cancer worldwide [3]. It is the fourth most common cancer among men in Taiwan, with an incidence of 7400 cases [4]. In Taiwan, it is the major malignancy in young adults, and the average age of death is lower than other cancers. In recent years, many innovative diagnostic and therapeutic approaches have been developed for the detection and treatment of oral cancer, significantly enhancing patient survival rates. Although, in some patients, due to the late diagnosis of the disease, the postoperative survival rate still needs to be improved. Early diagnosis and treatment of oral cancer are crucial for improving survival rate and achieving a better prognosis. During routine practice, clinicians identify oral cancer by conducting a traditional oral examination. If any abnormalities are observed, patients are recommended for a biopsy to establish a definitive diagnosis. However, biopsies are invasive procedures that may cause discomfort to patients and require both specialized skills and access to expensive laboratory resources, which are often only available in resource-rich settings. Hence, several non-invasive diagnostic methods have been developed at the clinical level to decrease the frequency of biopsies and enable early-stage diagnosis of oral cancer [5]. The existence of early detection techniques for oral cancer can aid in establishing a specialized area for accurate prognosis and prevention. Nonetheless, current diagnostic procedures and therapies have not yet achieved optimal effectiveness [6].

There are many non-invasive techniques, such as vital staining techniques [7], optical imaging, and autofluorescence imaging [8], to assist in the early detection of oral cancer. However,Raman spectroscopy is the most widely used optical technique for providing specific fingerprint-type information on molecules [9]. Before morphological changes in the diseased tissue take place, it can identify changes in the composition and content of biomolecules in a sample caused by cell proliferation, differentiation, or malignancy [10]. Raman spectroscopy presents numerous benefits over conventional chemical techniques, including eliminating the requirement for dilution and reagents, reduced analysis duration, provision of extensive information, and the ability to work with minimal sample volumes [11]. Multivariate analysis remains essential for pinpointing the most diagnostically relevant features within the spectral dataset and for delving deeper into the underlying biochemical alterations. The efficacy of Raman spectroscopy (an optical diagnostic technique) with multivariate statistical analyses was demonstrated using an excitation wavelength of 532 nm for oral cancer detection, using discriminant analysis (DA) followed by principal component analysis (PCA) and partial least squares (PLS) [12,13]. In a previous study [14], we explored the combined use of optical diagnostic techniques, namely Raman spectroscopy and VELscope, to enhance the detection rate of oral cancer. Since OSCC is commonly found among oral cancer patients, it is crucial to develop an accurate and non-invasive method for detecting OSCC. Although, at the early stage, adjuvant treatment can be more beneficial than in advanced stages. One global collaborative study underscores the significance of adjuvant therapy for early-stage OSCC, showing its potential to enhance patient outcomes and inform the creation of more efficient, tailored treatment approaches [15]. The potential of Raman spectroscopy has been repeatedly proven in many studies in discriminating OCSCC from non-tumorous tissue by using different techniques based on spectral data [16,17,18,19].

In this study, a Support Vector Machine (SVM) technique followed by PLS was proposed for classifying the OSCC from non-tumorous tissues, which could play an important role in the margin of resection for intraoperative use. Afterward, the cross-validation (CV) method was used to evaluate the model performance in terms of sensitivity, specificity, accuracy, precision, F1-measure (F1), balanced accuracy, Matthews correlation coefficient, and the receiver operating characteristic (ROC) curve.

## 2. Methods and Materials

### 2.1. Sample Collection and Preparation

The Chang Gung Memorial Hospital’s Institutional Review Board (IRB) approved this study through IRB No: 201800420B0 and 202300682B0. This study was conducted in the Department of Otolaryngology—Head and Neck Surgery in Taiwan. Tissue samples for ex vivo experiments with pathological reports were collected in the Chang Gung Memorial Hospital’s Department of Otolaryngology—Head and Neck Surgery. In this study, we measured 128 tissue samples taken from 64 patients (64 from adjacent sites of tumor and 64 from tumor cryopreserved tissue samples). The mean age of the patients was 55.4 ± 12.8 years and 92% of the patients were male. The patient demographics are illustrated in Table 1. Following surgery, participants were admitted to the ICU, where they provided their written informed consent for the collection of tissue samples. All samples measured a minimum of 3 × 3 mm. Surgical resection specimens from normal-looking mucosa near the tumor were collected 15–30 min after surgical removal, while tumor samples were acquired immediately post-surgery. The distance between the tumor border and the adjacent tissue (resection margin) is 1.5 to 2 cm. The examined cryopreserved samples were freshly sliced and stored in liquid nitrogen at −80 C to maintain their morphological integrity until use. Glass, being the most widely available substrate and providing satisfactory results at lower source wavelengths [20], was employed as the substrate for testing each tissue sample. From each tissue sample, five spectra were collected, resulting in a total of 320 spectra from tumor tissues and 320 spectra from non-tumorous parts. This provided a combined total of 640 spectra for analysis.

### 2.2. Raman Spectroscopy Detection and Data Acquisition

**Figure 1:**
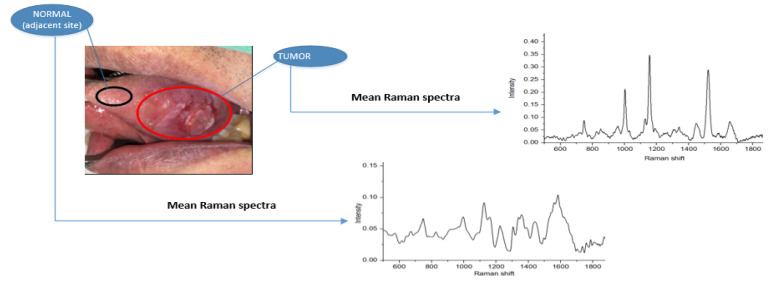
Sampling process: OCSCC of the tongue (tumor part) and normal sample taken from around 2 cm from tumor border along with mean Raman spectrum for normal and tumor specimen.

The Micro Raman Identify (MRI) system (ProTrusTech Co., Ltd.,Tainan, Taiwan) was used, which has a laser with an excitation wavelength of 532 nm and a laser power of 12.6 mW. The integration and acquisition durations were set at 5 s and 15 s, respectively, with an average spectrum value of 3. Before the analyses, the samples were removed from the refrigerator and allowed to thaw at room temperature for approximately 1 h. Subsequently, Raman spectra were recorded for the tissue samples at room temperature using the Raman spectroscopy technique. For each sample measurement, five spectra were gathered from the entire sample area, and the average of these five spectra was used for subsequent data analysis. Figure 1 displays a representative Raman spectrum with selected regions of interest (normal and tumor tissues) and their corresponding measured Raman spectra.

### 2.3. Data Analysis

Raman spectroscopy data were preprocessed to reduce interference, baseline correction, and eliminate data redundancy. After preprocessing, the spectral data consisted of 965 intensity variables, ranging from 500 cm${}^{-1}$ to 1800 cm${}^{-1}$. Spectral preprocessed data were analyzed using an SVM followed by PLS. To enhance the diagnostic performance of the SVM classifier, dimensionality reduction of the tissue’s Raman spectral data was required. PLS was employed to extract features from the pre-processed Raman spectra of the tissue samples. The mean-normalized spectral data were analyzed using the PLS–SVM, developed in MATLAB (R2018a, MathWorks, MA, USA), which is a multivariate statistical method. The main rationale for employing this multivariate statistical model is that the PLS method eliminates redundancy and noise in high-dimensional datasets, while also enabling the reduced-dimension features to serve as input for the SVM classifier [21]. The PLS model was employed to estimate the concentrations of various biomolecules, such as proteins, nucleic acids, lipids, and more, to their respective Raman spectra. In PLS discriminant analysis, the latent variables (LVs) are oriented to maximize group differentiation. Consequently, the LVs evaluate diagnostically relevant alterations rather than substantial variations within the dataset [22]. SVM has been demonstrated to be an effective technique for carrying out nonlinear classification, multivariate function estimation, or nonlinear regression. It efficiently prevents overfitting and underfitting issues, resulting in a more efficient and convincing outcome [23].

## 3. Results

A total of 128 tissue samples were analyzed using Raman spectroscopy. Among these samples, 90 were utilized to develop the OSCC detection algorithm or classification model with the training dataset. The remaining 38 samples served to validate the model using the testing dataset. The tissue samples were collected from 64 patients diagnosed with oral cancer, with 64 samples from non-tumorous regions and 64 from tumor lesions. Within the OSCC patient group, 6 patients were in the first stage (T1), 17 in the second stage (T2), 10 in the third stage (T3), and 31 in the fourth stage (T4).

### 3.1. Spectral Features

**Figure 2:**
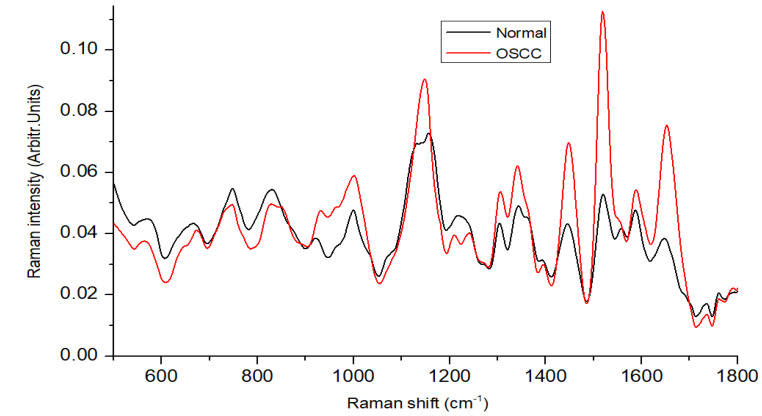
Mean Raman spectra of oral normal and OSCC tissues.

Figure 2 depicts the average Raman spectra for the 64 non-tumorous and 64 OSCC patients after pre-processing, within the wave-number range of 500–1800 cm${}^{-1}$. In our study, we found that the average spectra of non-tumorous tissue exhibited lower peaks compared to OSCC, with major contributions from proteins, nucleic acids, amino acids, carbohydrates, and lipids. The literature confirms that spectral peaks in normal tissue are primarily influenced by lipids, while malignant tissue peaks are predominantly driven by proteins [13,24]. The spectra of malignant or tumor tissue samples were characterized by peaks at 1004, 1156, 1339, 1450, 1523, and 1655 cm${}^{-1}$, while normal tissue samples exhibited dominant peaks at 747,829, 1072, 1220, and 1734 cm${}^{-1}$. It can be observed that both types of tissues exhibit carotenoid peaks at 1523 cm${}^{-1}$ and 1155 cm${}^{-1}$, with tumorous tissues displaying more intense carotenoid peaks compared to normal tissue. In one study [25], these peaks (1004, 1156, and 1523 cm${}^{-1}$) were observed in the carcinoma Raman patterns during the investigation, and they were significantly less intense in healthy tissues, which is in good agreement with our results. The presence of peaks at 1156 and 1518 cm${}^{-1}$ in our analysis resulted from mixed signals, with contributions from both carotenoids and proteins. The peak at 1004 cm${}^{-1}$ can be attributed to the characteristic phenylalanine of proteins [26]. The Raman bands for phospholipids at 1078 and 1745 cm${}^{-1}$, as well as tyrosine at 823 cm${}^{-1}$, exhibit a significant reduction in normalized intensity, suggesting a lower proportion of phospholipids in relation to the total Raman-active constituents in tumor tissue [27]. The peak at 1078 cm${}^{-1}$ in normal tissue, attributed to the C–C or C–O stretching mode of phospholipids, nearly disappears in tumor tissue, indicating reduced vibrational stability of lipid chains in tumors [28]. Cancerous spectral characteristics consist of a wide and pronounced amide I peak at 1655 cm${}^{-1}$, a CH2 bending peak at 1450 cm${}^{-1}$, broad peaks in the amide III range of 1265–1350 cm${}^{-1}$, and the distinct phenylalanine peak at 1004 cm${}^{-1}$, all suggesting a significant protein contribution. Meanwhile, in normal tissue, the sharp, weak band at 1655 cm${}^{-1}$ is attributed to the C=C bonds of lipids, rather than amide I. The smaller peak at 1745 cm${}^{-1}$ in normal tissue indicates the lipid assignment of phospholipids. A broad peak at 1220 cm${}^{-1}$ indicates a lipid assignment attributed to the =CH bending. The scores of factors are linear combinations of original variables that capture the maximum variance in the dataset while reducing dimensionality. The scores of components offer quantitative insights into the biomolecules available, and this information can be utilized to distinguish between the different groups in a large dataset.

### 3.2. Optimization of PLS Components and Evaluation

**Figure 3:**
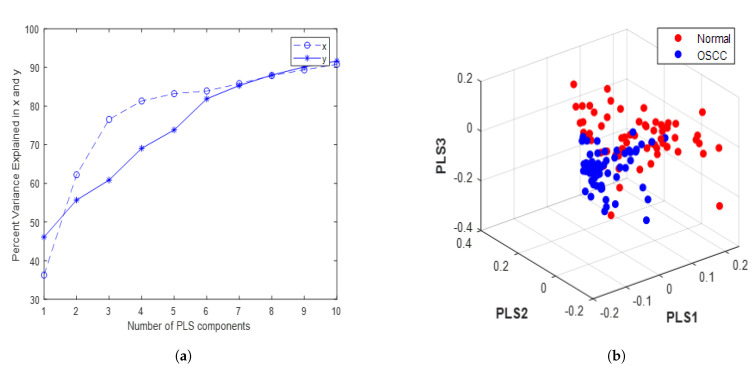
PLS. (**a**) Variance of x (predictors) and y (response variable) vs. the number of PLS components in the tissue sample dataset; (**b**) 3D scatter plot.

The classifier models were optimized using a training dataset to assess the classification results, and their performance was subsequently evaluated using a separate test dataset. The optimal number of PLS components is typically chosen based on the performance measures obtained through the K-fold cross-validation (KFCV) method [29]. KFCV was used to assess the prediction performance of PLS models with different numbers of components, finally, it provides the optimal number of components that minimizes the average prediction error (mean squared error) or provides the highest coefficient of determination (R-squared) across the k-folds. The R-squared value and mean squared error were 0.9166 and 0.1048 for 10 PLS components, respectively. k-fold cross-validation usually provides an unbiased estimate of the true model accuracy; a larger validation value is recommended due to the good balance between bias and variance [30]. The first 10 components had a strong correlation coefficient of 0.95 between the X and Y scores in the PLS model. These PLS components were fed into the SVM classifier, and the analysis primarily classified the patients into two groups: with OSCC and without OSCC. Table 2 illustrates the relationship between the number of PLS components and the corresponding computation time needed to evaluate the model’s performance. The number of latent variables (components) increased with computation time [31]. Computation time was calculated by running the PLS function multiple times and calculating the average computation time. The initial “warm-up” run was executed before measuring the time. Once 10 PLS components were employed, the graph revealed that the explained variance for both predictors and the response variable reached a saturation point, with a negligible increase beyond that (Figure 3a). Figure 3b shows a scatter plot between the first three PLS components, which can be used to visualize the relationships and patterns among the data points in a reduced-dimensionality space. A ROC curve was employed to illustrate the classification of tissue samples into non-OSCC and OSCC groups by plotting the sensitivity (true positive rate) against the false positive rate (1-specificity).

### 3.3. Training and Validation Procedure of SVM Model

**Figure 4:**
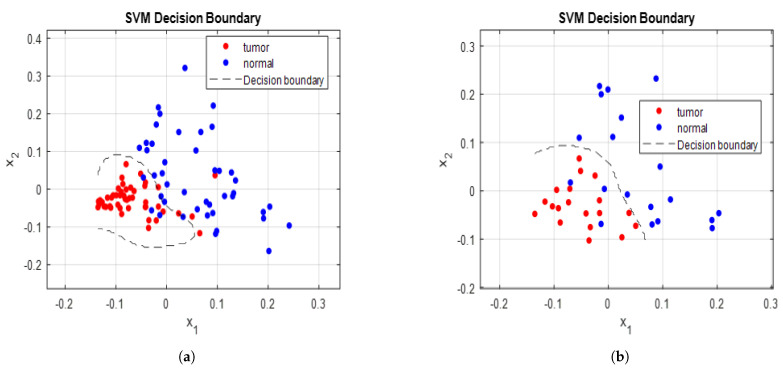
SVM decision boundary curve for (**a**) training data, (**b**) testing data.

Dividing the dataset into training and testing subsets is a crucial step in evaluating the performance of a machine learning model. In our process, we split the dataset into two parts: one for training the model (70% of the data) and the other for testing its performance (30% of the data). This approach helps to assess the model’s ability to generalize to unseen data, reducing the risk of over-fitting. To divide the dataset, we randomly shuffled the data and then allocated 70% of the samples for training and the remaining 30% for testing. This process ensures that both subsets have a representative distribution of the original data. To further improve the reliability of the performance evaluation, we performed the splitting procedure multiple times, each time creating a new training and testing set. This approach is called repeated random sampling [32]. By averaging the performance metrics (e.g., accuracy, precision, sensitivity) over multiple iterations, we can obtain a more accurate and robust estimate of the model’s performance. Figure 4 displays the 2D boundary plot for the SVM classifier after processing by PLS. The 2D boundary curve for the SVM classifier provides a visual representation of the decision boundary that the algorithm uses to distinguish between different classes in a two-dimensional feature space. The curve was generated by considering the support vectors, which are the data points closest to the decision boundary, and the margin, which represents the distance between the support vectors and the decision boundary. The SVM algorithm aims to maximize this margin to improve classification performance and robustness. Figure 4a represents the plot for the training dataset, while Figure 4b illustrates the plot for the testing dataset. The decision boundary effectively distinguishes between the two classes in this data.

### 3.4. Evaluation of Model Using Testing Set

To ensure the reliability and generalizability of the SVM classifier’s performance, k-fold cross-validation was employed during the model evaluation process. In k-fold cross-validation, the dataset is divided into k equally sized folds. The model is then trained on k-1 folds and tested on the remaining fold. This process is repeated k times, with each fold being used as the test set exactly once. The average performance metric, such as accuracy, precision, recall, or F1-score, is calculated by aggregating the results from all k iterations. By using k-fold cross-validation, we can obtain a more robust estimate of the SVM classifier’s performance, minimizing the risk of overfitting and providing a better understanding of how the model is expected to perform on unseen data. The tissue Raman spectra from cancer patients, including both the operated tumor regions and adjacent normal sites, were analyzed to develop a differential diagnosis model. The performance table of the SVM classifier using testing data is shown in Table 3. The model’s performance was assessed using various evaluation metrics, such as sensitivity, specificity, accuracy, F1-score, balanced accuracy, Matthews correlation coefficient, and area under the curve (AUC). Cross-validation was employed as the method for evaluating the model, ensuring a robust and reliable assessment of its diagnostic capabilities. In the tissue sample analysis, we obtained an accuracy of 94.7% for distinguishing between the squamous carcinoma tissue and the normal tissue. The sensitivity and specificity were 95.7% and 93.3%, respectively. These results suggest that the diagnostic model is stable and can be effectively employed for identifying tissue samples from patients at various stages of disease.

**Figure 5:**
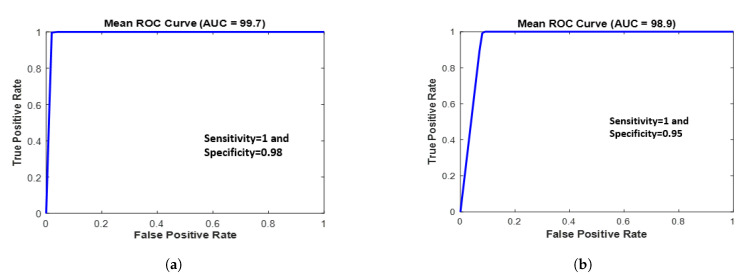
ROC curve of the classification results for the PLS–SVM model using (**a**) training data and (**b**) testing data.

A PLS–SVM model was used to analyze the statistical efficiency of this tool. PLS is a dimensionality reduction technique used to extract the most relevant features from the data, while SVM is a supervised machine learning algorithm used for classification or regression tasks. The PLS data were calculated for tumorous and normal tissue in the fingerprint region 500–1800 cm${}^{-1}$. The spectral data were analyzed according to the first ten PLS components. The PLS–SVM model is preferable to spectral data and is the most effective multivariate statistical technique [33]. In this study, an ROC curve was used to evaluate the clinical potential of the PLS–SVM model, with the AUC offering an estimation of sensitivity and specificity at various significance levels. The ROC curve was generated using both training and testing datasets. The training dataset yielded an accuracy of 99% with 99.7% AUC, while the testing dataset provided an accuracy of 97.5% with 98.9% AUC, as exhibited in Figure 5. These results suggest that the classification model has great potential for accurate and reliable diagnosis in clinical settings. However, further validation with larger datasets is recommended to ensure the robustness and applicability of the model in real-world scenarios. According to the model, most of the biomolecular information from tissue samples is vital for distinguishing OSCC tissue from healthy tissue. All tissue samples in this study were obtained from oral surgery patients, and the goal was to achieve higher sensitivity and specificity using the PLS–SVM classification model.

## 4. Discussion

Tissue samples can provide crucial insights into the molecular, cellular, and histological features of various diseases, which can aid in the development of novel diagnostic tools, therapies, and prevention strategies. By investigating the molecular, cellular, and histological features of tissue samples, researchers can gain important insights into disease mechanisms, identify novel diagnostic and therapeutic targets, and evaluate the effectiveness of treatments. In this study, we used the cryopreserved technique to preserve the tissue samples. Cryopreserved tissue samples play a crucial role in biomedical research and diagnostic applications. The process of cryopreservation involves preserving tissue samples at extremely low temperatures, typically using liquid nitrogen or ultra-low freezers, to maintain their structural integrity, molecular composition, and biological function. this also minimizes the degradation of cellular components and biomolecules, such as DNA, RNA, proteins, and metabolites, ensuring that the samples remain representative of their original state. This preservation of biological information is vital for accurate data analysis and interpretation.

The Raman spectra of OSCC specimens revealed significantly higher nucleic acid, protein, carotenoids, and various amino acid contents compared to adjacent healthy tissues. Conversely, the Raman spectra of healthy specimens showed a significantly higher lipid content than tumorous tissue. The substantial differences between cancerous and healthy tissues led to a high discrimination rate in this study, even when using a testing dataset with the cross-validation technique, which is a fair approach for assessing model performance. One possible reason for these findings is that most cancer patients in this study were at an advanced stage, resulting in more significant variations between the two types of spectra.

**Figure 6:**
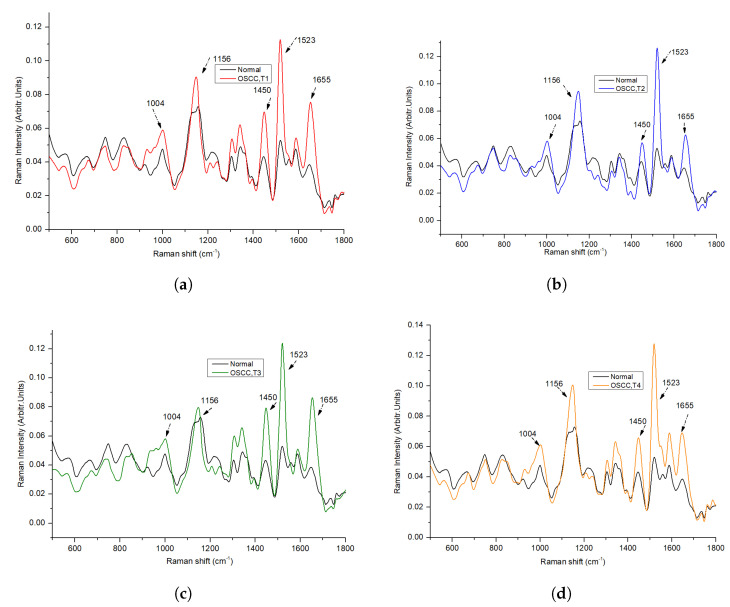
Comparison of Average Raman Spectra Between Normal Tissue and Various Stages of OSCC: (**a**) Normal vs. Stage 1 OSCC (T1), (**b**) Normal vs. Stage 2 OSCC (T2), (**c**) Normal vs. Stage 3 OSCC (T3), and (**d**) Normal vs. Stage 4 OSCC (T4).

We have demonstrated that Raman spectroscopy is a useful tool for diagnosing OSCC from tissue samples. Despite the limited number of oral cancer patients included in this study, we were able to differentiate between various stages of the disease solely using Raman spectroscopy, without resorting to any classifier models. The mean Raman spectra of each stage, when compared with normal tissues as depicted in Figure 6, provides initial insight into stage-specific variations. This represents our preliminary results towards a stage-biased classification approach. In Figure 6, we observed that the protein and carotenoid peaks (at 1156 and 1523 cm${}^{-1}$, respectively) were more pronounced in the stage 4 OSCC tissue samples compared to others. However, stage 3 spectra seemed to deviate from this trend. This inconsistency could potentially be due to dominant subsites, as different stages of the disease manifest in different subsites [34]. Similarly, the Amide I peak at 1655 cm${}^{-1}$ was more prominent in stage 1 and stage 3 samples than in stages 2 and 4. This observation aligns with our finding that most tissue samples from stages T1 and T3 originate from the buccal mucosa subsite, which characteristically presents a more intense peak than tongue and gingiva subsites [12]. The phenylalanine peak, situated at 1004 cm${}^{-1}$, exhibited the highest intensity in stage 4 cancer. The CH2 bending peak at 1450 cm${}^{-1}$ showed higher intensity in stage 3 patients. From these observations, it became clear that when classifying OSCC by stages, it is crucial to consider subsites as well. As we expand our tissue sample collection, future research could delve deeper into the impact of different subsites on OSCC stages using Raman spectroscopy.

Despite significant advancements in the medical field, emerging diseases and complications continue to pose considerable threats to human life. While our understanding of the complexities associated with OSCC is more extensive compared to novel diseases, treatment may sometimes be delayed due to late detection or diagnostic errors, leading to severe consequences or even patient mortality. By employing Raman spectroscopy, the rapid analysis of biomolecules present in tissue samples becomes possible, potentially reducing the occurrence of such delays and improving patient outcomes. The use of Raman spectroscopy in clinical practice is an area of active research and development. While it is not yet a widely established clinical tool, there is growing interest in its potential applications. Researchers are investigating the integration of Raman spectroscopy into clinical workflows to enhance diagnostic accuracy, enable real-time tumor margin assessment during surgery, and provide valuable information for personalized treatment decisions.

Raman spectroscopy is advantageous in surgical applications due to its high accuracy and rapid analysis capabilities. It can be utilized to differentiate between healthy and cancerous tissues during surgery, ensuring more precise tumor resection and improved surgical outcomes. In oral cavity cancer, obtaining adequate surgical margins can sometimes be challenging; however, Raman analysis could be particularly helpful in this aspect by providing real-time feedback on tissue composition, enabling surgeons to make informed decisions and minimize the risk of leaving behind residual cancerous cells.

## 5. Conclusions

This study introduces a non-invasive, fast, and straightforward method for identifying healthy tissue adjacent to tumors in OSCC patients. The observed spectral differences between healthy and OSCC patients’ tissues could be attributed to alterations in the distribution and conformation of tissue metabolites, including amino acids, nucleotides, sugars, lipids, and organic acids. The PLS–SVM analysis of the spectral data yielded an accuracy of 94.7%, sensitivity of 95.7%, and specificity of 93.3%. Future research should expand the sample size to validate these preliminary findings and aim to delve deeper into how different subsites influence OSCC’s Raman spectra. This methodical approach will improve OSCC diagnostic precision, possibly directing customized treatment plans for varying OSCC stages and subsites to facilitate the early diagnosis of oral cancer.

## Figures and Tables

**Table 1:** Patient Demographics Table.

Characteristics		Age (Mean ± SD)		Gender (M:F)	
		55.4 ± 12.8		59:5	
**Location**	Tongue	14 (21.9%)			
	Mouth floor	5 (7.8%)			
	Lip	2 (3%)			
	Buccal mucosa	28 (43.8%)			
	Alveolus (gum)	14 (21.9%)			
	Retromolar trigone	1 (1.6%)			
**Tumor Stage**		T1	T2	T3	T4
		6 (9.5%)	17 (26.3%)	10 (15.7%)	31 (48.5%)

**Table 2:** Computation Time (in milliseconds) for PLS-SVM Classifier with Varying PLS Components.

Number of PLS Components	Computation Time (in msec) with Classifier
2	7.1
5	7.6
10	9.9

**Table 3:** Performance table of SVM classifier: SEN (sensitivity), SPE (specificity), AC (accuracy), PRE (precision), F1 (F1-score), BAC (balanced accuracy), MCC (Matthews correlation coefficient).

PLS-SVM	SEN	SPE	AC	PRE	BAC	F1-Score	MCC
**Parameters**	95.65	93.33%	94.74%	95.65%	94.49%	95.65%	0.889

## Data Availability

The data sets generated for this study are available on request from the corresponding author.

## References

[1] Rai P., Goh C.E., Seah F., Islam I., Chia-Wei W.W., Mcloughlin P.M., Loh J.S.P. Oral Cancer Awareness of Tertiary Education Students and General Public in Singapore. Int. Dent. J..

[2] Chi A.C., Day T.A., Neville B.W. (2015). Oral cavity and oropharyngeal squamous cell carcinoma—An update. CA Cancer J. Clin..

[3] Sung H., Ferlay J., Siegel R.L., Laversanne M., Soerjomataram I., Jemal A., Bray F. (2021). Global cancer statistics 2020: GLOBOCAN estimates of incidence and mortality worldwide for 36 cancers in 185 countries. CA Cancer J. Clin..

[4] Jhuang J.R., Su S.Y., Chiang C.J., Yang Y.W., Lin L.J., Hsu T.H., Lee W.C. (2022). Forecast of peak attainment and imminent decline after 2017 of oral cancer incidence in men in Taiwan. Sci. Rep..

[5] Bhatia N., Lalla Y., Vu A.N., Farah C.S. (2013). Advances in optical adjunctive AIDS for visualisation and detection of oral malignant and potentially malignant lesions. Int. J. Dent..

[6] Chakraborty D., Ghosh D., Kumar S., Jenkins D., Chandrasekaran N., Mukherjee A. (2023). Nano-diagnostics as an emerging platform for oral cancer detection: Current and emerging trends. Wiley Interdiscip. Rev. Nanomed. Nanobiotechnol..

[7] Liu D., Zhao X., Zeng X., Dan H., Chen Q. (2016). Non-invasive techniques for detection and diagnosis of oral potentially malignant disorders. Tohoku J. Exp. Med..

[8] Jeng M.J., Sharma M., Chao T.Y., Li Y.C., Huang S.F., Chang L.B., Chow L. (2020). Multiclass classification of autofluorescence images of oral cavity lesions based on quantitative analysis. PLoS ONE.

[9] Pence I., Mahadevan-Jansen A. (2016). Clinical instrumentation and applications of Raman spectroscopy. Chem. Soc. Rev..

[10] Cordero E., Latka I., Matthäus C., Schie I.W., Popp J. (2018). In-vivo Raman spectroscopy: From basics to applications. J. Biomed. Opt..

[11] Saatkamp C.J., de Almeida M.L., Bispo J.A.M., Pinheiro A.L.B., Fernandes A.B., Silveira L. (2016). Quantifying creatinine and urea in human urine through Raman spectroscopy aiming at diagnosis of kidney disease. J. Biomed. Opt..

[12] Jeng M.J., Sharma M., Sharma L., Chao T.Y., Huang S.F., Chang L.B., Wu S.L., Chow L. (2019). Raman spectroscopy analysis for optical diagnosis of oral cancer detection. J. Clin. Med..

[13] Sharma M., Jeng M.J., Young C.K., Huang S.F., Chang L.B. (2021). Developing an Algorithm for Discriminating Oral Cancerous and Normal Tissues Using Raman Spectroscopy. J. Pers. Med..

[14] Jeng M.J., Sharma M., Sharma L., Huang S.F., Chang L.B., Wu S.L., Chow L. (2020). Novel Quantitative Analysis Using Optical Imaging (VELscope) and Spectroscopy (Raman) Techniques for Oral Cancer Detection. Cancers.

[15] Fridman E., Na’ara S., Agarwal J., Amit M., Bachar G., Villaret A.B., Brandao J., Cernea C.R., Chaturvedi P., Clark J. (2018). The role of adjuvant treatment in early-stage oral cavity squamous cell carcinoma: An international collaborative study. Cancer.

[16] Wang R., Wang Y. (2021). Fourier transform infrared spectroscopy in oral cancer diagnosis. Int. J. Mol. Sci..

[17] Chen P.H., Shimada R., Yabumoto S., Okajima H., Ando M., Chang C.T., Lee L.T., Wong Y.K., Chiou A., Hamaguchi H.o. (2016). Automatic and objective oral cancer diagnosis by Raman spectroscopic detection of keratin with multivariate curve resolution analysis. Sci. Rep..

[18] Dai W.Y., Lee S., Hsu Y.C. (2018). Discrimination between oral cancer and healthy cells based on the adenine signature detected by using Raman spectroscopy. J. Raman Spectrosc..

[19] Knipfer C., Motz J., Adler W., Brunner K., Gebrekidan M.T., Hankel R., Agaimy A., Will S., Braeuer A., Neukam F.W. (2014). Raman difference spectroscopy: A non-invasive method for identification of oral squamous cell carcinoma. Biomed. Opt. Express.

[20] Kerr L.T., Byrne H.J., Hennelly B.M. (2015). Optimal choice of sample substrate and laser wavelength for Raman spectroscopic analysis of biological specimen. Anal. Methods.

[21] Jeng M.J., Sharma M., Lee C.C., Lu Y.S., Tsai C.L., Chang C.H., Chen S.W., Lin R.M., Chang L.B. (2022). Raman Spectral Characterization of Urine for Rapid Diagnosis of Acute Kidney Injury. J. Clin. Med..

[22] Liu W., Sun Z., Chen J., Jing C. (2016). Raman spectroscopy in colorectal cancer diagnostics: Comparison of PCA-LDA and PLS-DA models. J. Spectrosc..

[23] Liu D., Guo W. (2015). Identification of kiwifruits treated with exogenous plant growth regulator using near-infrared hyperspectral reflectance imaging. Food Anal. Methods.

[24] Guze K., Pawluk H.C., Short M., Zeng H., Lorch J., Norris C., Sonis S. (2015). Pilot study: Raman spectroscopy in differentiating premalignant and malignant oral lesions from normal mucosa and benign lesions in humans. Head Neck.

[25] Rau J.V., Fosca M., Graziani V., Taffon C., Rocchia M., Caricato M., Pozzilli P., Onetti Muda A., Crescenzi A. (2017). Proof-of-concept Raman spectroscopy study aimed to differentiate thyroid follicular patterned lesions. Sci. Rep..

[26] Malini R., Venkatakrishna K., Kurien J., Pai K.M., Rao L., Kartha V., Krishna C.M. (2006). Discrimination of normal, inflammatory, premalignant, and malignant oral tissue: A Raman spectroscopy study. Biopolym. Orig. Res. Biomol..

[27] Movasaghi Z., Rehman S., Rehman I.U. (2007). Raman spectroscopy of biological tissues. Appl. Spectrosc. Rev..

[28] Huang Z., McWilliams A., Lui H., McLean D.I., Lam S., Zeng H. (2003). Near-infrared Raman spectroscopy for optical diagnosis of lung cancer. Int. J. Cancer.

[29] Abdi H. (2010). Partial least squares regression and projection on latent structure regression (PLS Regression). Wiley Interdiscip. Rev. Comput. Stat..

[30] Kohavi R. A study of cross-validation and bootstrap for accuracy estimation and model selection. Proceedings of the IJCAI’95: Proceedings of the 14th International Joint Conference on Artificial Intelligence, Montreal, QC, Canada, 20–25 August 1995.

[31] Geladi P., Kowalski B.R. (1986). Partial least-squares regression: A tutorial. Anal. Chim. Acta.

[32] Gareth J., Daniela W., Trevor H., Robert T. (2013). An Introduction to Statistical Learning: With Applications in R.

[33] Wang K., Qiu Y., Wu C., Wen Z.N., Li Y. (2023). Surface-enhanced Raman spectroscopy and multivariate analysis for the diagnosis of oral squamous cell carcinoma. J. Raman Spectrosc..

[34] Carvalho L.F.C., Nogueira M.S., Bhattacharjee T., Neto L.P., Daun L., Mendes T.O., Rajasekaran R., Chagas M., Martin A.A., Soares L.E.S. (2019). In vivo Raman spectroscopic characteristics of different sites of the oral mucosa in healthy volunteers. Clin. Oral Investig..

